# Whole‐exome sequencing analyses in a Saudi Ischemic Stroke Cohort reveal association signals, and shows polygenic risk scores are related to Modified Rankin Scale Risk

**DOI:** 10.1007/s10142-023-01039-7

**Published:** 2023-03-27

**Authors:** Fahad A. Alkhamis, Majed M. Alabdali, Abdulla A. Alsulaiman, Abdullah S. Alamri, Rudaynah Alali, Mohammed S. Akhtar, Sadiq A. Alsalman, Cyril Cyrus, Aishah I. Albakr, Anas S. Alduhalan, Divya Gandla, Khaldoun Al-Romaih, Mohamed Abouelhoda, Bao-Li Loza, Brendan Keating, Amein K. Al-Ali

**Affiliations:** 1 Department of Neurology, King Fahd Hospital of The University, Imam Abdulrahman Bin Faisal University, Dammam, 31441 Saudi Arabia; 2 Department of Internal Medicine, King Fahd Hospital of The University, Imam Abdulrahman Bin Faisal University, Dammam, 31441 Saudi Arabia; 3 Department of Clinical Biochemistry, College of Medicine, Imam Abdulrahman Bin Faisal University, Dammam, 31441 Saudi Arabia; 4 Department of Neurology, King Fahd Hospital, Alhafof, Saudi Arabia; 5 Department of Surgery, Perelman School of Medicine, University of Pennsylvania, Pennsylvania, PA 19104 USA; 6 Centre for Genomic Medicine, KFSH&RC, Riyadh, 11211 Saudi Arabia

**Keywords:** Exome sequencing, Stroke, Saudi, Variants, Dominant, Recessive

## Abstract

Ischemic stroke represents a significant societal burden across the globe. Rare high penetrant monogenic variants and less pathogenic common single nucleotide polymorphisms (SNPs) have been described as being associated with risk of diseases. Genetic studies in Saudi Arabian patients offer a greater opportunity to detect rare high penetrant mutations enriched in these consanguineous populations. We performed whole exome sequencing on 387 ischemic stroke subjects from Saudi Arabian hospital networks with up to 20,230 controls from the Saudi Human Genome Project and performed gene burden analyses of variants in 177 a priori loci derived from knowledge-driven curation of monogenic and genome-wide association studies of stroke. Using gene-burden analyses, we observed significant associations in numerous loci under autosomal dominant and/or recessive modelling. Stroke subjects with modified Rankin Scale (mRSs) above 3 were found to carry greater cumulative polygenic risk score (PRS) from rare variants in stroke genes (standardized PRS mean > 0) compared to the population average (standardized PRS mean = 0). However, patients with mRS of 3 or lower had lower cumulative genetic risk from rare variants in stroke genes (OR (95%CI) = 1.79 (1.29–2.49), *p* = 0.0005), with the means of standardized PRS at or lower than 0. In conclusion, gene burden testing in Saudi stroke populations reveals a number of statistically significant signals under different disease inheritance models. However, interestingly, stroke subjects with mRS of 3 or lower had lower cumulative genetic risk from rare variants in stroke genes and therefore, determining the potential mRS cutoffs to use for clinical significance may allow risk stratification of this population.

**Supplementary Information:**

The online version contains supplementary material available at 10.1007/s10142-023-01039-7.

## Introduction

Stroke is a major cause of morbidity and is the second leading cause of death worldwide. Over 13 million people have a stroke each year and around 5.5 million people will die as a result (Virani et al. 2021). Incidence, type of stroke and mortality rates vary markedly between countries and ancestral groups. Ischemic strokes are the most common type of stroke, and typically involve a disruption of blood flow to the brain parenchyma which causes brain cell death from a lack of oxygen. It may result from a number of processes including small vessel occlusions (SVOs) and large-artery atherosclerosis (Adams et al. 1993). More young people are affected with ischemic stroke in low- and middle-income countries than higher income countries (Lehman and Fullerton 2013; Lehman et al. 2018). Initial twin and family studies, primarily using linkage analysis, contributed to the initial knowledge of heritability studies in stroke (Flossmann et al. 2004). While such approaches found causal variants in various genes for monogenic stroke disorders, they had limited value in finding common variants impacting polygenic risk (Ekkert et al. 2021; Li et al. 2021). Genome-Wide Association Studies (GWAS) utilizing genome-wide genotyping arrays and/or whole exome sequencing (WES) have been successful in elucidating rare and common variants in various stroke subtypes (Li et al. 2021; Dichgans et al. 2021; Kumar et al. 2021).

Polygenic risk scores (PRS) utilizing cumulative, weighted risk scores for multiple genetic variants, with specific diseases/phenotypes, typically integrate known clinical risk covariates. They have been used with varying success in common diseases with multifactorial disease risk, including those with common and rare genetic underpinnings. Malik and colleagues combined stroke PRS data with Framingham risk scores and observed a significant association with ischemic stroke risk, although the prognostic value of the PRS was not substantially different from that of conventional clinical risk factors (Malik et al. 2014). However, more recent studies have shown utility with the use of PRSs in stroke-related phenotypes (Hachiya et al. 2020).

Saudi Arabia has seen an unprecedented adverse rise in modifiable risk factors for vascular disease over the last 30–40 years including poor diet, smoking and sedentary lifestyle, resulting in increases in dyslipidemia, type 2 diabetes, and hypertension which further exacerbate stroke and other vascular disease progression (Alhazzani et al. 2021). There are wide incidence differences across reported Saudi stroke studies ranging from ~ 16 to 58 cases per 100,000 person years (Alhazzani et al. 2018; Al Rajeh & Awada 2002). Such differences may be due to an interplay of study ascertainment biases as well as from significant primary and secondary health care delivery differences between private and public payer systems in Saudi Arabia, leading to a higher likelihood of undiagnosed diseases (Alqahtani et al. 2020). Recent studies indicate that the incidence of stroke is increasing rapidly with ischemic stroke being the dominant subtype affecting the Saudi populations (Alhazzani et al. 2018; Alqahtani et al. 2020; Al Rajeh & Awada 2002; Alqahtani et al. 2020).

Performing genetic studies in Saudi Arabian populations offers a unique opportunity for the discovery of novel genetic variants impacting disease risk due to a high rate of consanguinity amongst tribal pedigrees that make up the majority of the national population (Kari et al. 2014; Alkuraya 2012). We performed whole exome sequencing on 387 Saudi subjects with clinically diagnosed ischemic stroke. We focused on the analyses of exonic sequence data from a panel of 177 gene regions derived from highly curated stroke studies and utilized approximately 20,230 controls from the Saudi Human Genome Project. We then assessed the association of rare variants, primarily with ischemic stroke, and also evaluated PRSs within our study population.

## Methods

### Study participants’ samples and data

During 2019–2020, samples and data from 387 subjects (inpatients and outpatients) who had been diagnosed with ischemic stroke and attending the following Neurology Clinics were collected for inclusion in this study: King Fahd Hospital of the University (KFHU), Al Khobar; King Fahd Hospital, Al Hafof; and Al Wajh Hospital, Dammam. Participants ranged in age from 19 to 81. The phenotype data of all subjects were reviewed by a neurology consultant to ascertain and verify the diagnoses and the phenotype uniformity among sites as well as eligibility according to the study criteria. Table 1 outlines the demographic and clinical characteristics of the 387 ischemic stroke subjects included in this study. The subtypes of ischemic stroke were determined according to the Trial of Org 10,172 in Acute Stroke Treatment (TOAST) classification (Adams et al. 1993). The functional outcome of stroke patients was determined using modified Rankin Scale (mRS) at admission and at one-month post stroke follow-up.

**Table 1:** Saudi Ischemic Stroke Study Participants Clinical Characteristics. Clinical characteristics data collated from 387 ischemic stroke subjects diagnosed at neurology clinics at the following three Saudi Hospitals: King Fahd Hospital University (KFHU), Al-Khobar, and King Fahd Hospital, Alhafof and Al Wajh Hospital, Dammam. *Trial of Org 10,172 in Acute Stroke Treatment (TOAST) classification

Parameter	
Total number of patients	387
Age (Mean ± SD)	56.5 ± 15.8
Age at diagnosis (Mean ± SD)	55.6 ± 15.7
Male sex *N* (%)	223 (57.6)
Hospital	
AL-WAJAH, Dammam, *n* = (%)	94 (24.3)
King Fahd Hospital, Al Hafof, *n* = (%)	107 (27.6)
KFHU, Alkhobar, *n* = (%)	186 (48.1)
TOAST* classification	
Large artery atherosclerosis, *n* = (%)	78 (20.2)
Cardio aortic embolism, *n* = (%)	56 (14.5)
Small artery occlusion, *n* = (%)	253 (65.4)
Modified Rankin Scale (mRS)	
0 N (%)	51 (13.2)
1 N (%)	44 (11.4)
2 N (%)	219 (56.6)
3 N (%)	38 (9.8)
4 N (%)	17 (4.4)
5 N (%)	12 (3.1)
6 N (%)	6 (1.6)

### DNA extraction and sequencing

Peripheral blood samples were collected into EDTA tubes and stored in − 20 °C freezers at the research laboratories at the College of Medicine, Imam Abdulrahman bin Faisal University. Genomic DNA extraction from all samples was performed using Gentra Puregene Blood kits (Qiagen, USA) according to the manufacturer’s protocol. Whole exome sequencing libraries were generated using the SureSelect Human All Exon Kit v5 (Agilent, CA, USA) and sequenced on a HiSeq instrument (Illumina, CA, USA) using a standard paired-end sequencing protocol for SureSelectXT Library Prep and Target Enrichment System Version B.2 (Illumina, CA, USA).

### Read alignment, variant calling, and QC

Reads in the FASTQ files were aligned to the standard human genome reference (GRCh37) using Illumina’s Dynamic Read Analysis dor GENNomics (DRAGEN) Genomic Pipeline. Resultant BAM files were position-sorted and duplicate reads marked. Single-sample genomic variant call files (gVCF) were generated by the DRAGEN Germline Pipeline, and joint calling of all samples in the study cohort was performed by DRAGEN Joint Genotyping (Illumina, CA, USA).

### Principal components analysis (PCA) and Kinship

The *KING* algorithm was used for relatedness inference based on the genotype of exome SNPs (minor allele frequency [MAF] > 0.01). Estimated kinship coefficient and number of SNPs with zero shared alleles (identity by state [IBS]0) between a pair of individuals were plotted. Parent-offspring, sibling pairs, and unrelated pairs were visualized on the scatterplot to distinguish any separate clusters. Ancestry and Kinship Toolkit (AKT) was used to calculate PCAs and plot the results using 1000 genome project data. The study participant samples demonstrated a genetically matched background consistent with typical Saudi populations including African admixture, which is known to be evident in Saudi tribes primarily from East Africa (Fernandes et al. 2019).

### Variant annotation, filtering and prioritization

Variants were annotated with a program for annotating and predicting the effects of single nucleotide polymorphisms (SnpEff,v5.0) to predict the effects of the variants. Rare variants were defined as minor allele frequency (MAF) < 1% in The Genome Aggregation Database (gnomAD) (v2.1.1). Intronic, synonymous, 3′ and 5′ UTR, upstream and downstream variants were identified and excluded from the analysis. The remaining rare variants were considered to be potentially deleterious variants. Genetic variants classified in ClinVar as “Likely pathogenic” or “Pathogenic,” and in Human Gene Mutation Database (HGMD) as disease-causing mutations (DM) for stroke were collected and curated together with research literature to serve as the knowledgebase for variant prioritization and classification (Stenson et al. 2003).

### Use of a comprehensive stroke gene panel

Numerous reports have identified genes associated with stroke by using data from monogenic and genome-wide association studies (GWAS). We used a comprehensive collation of genes with associations for monogenic causes of stroke which has been used in many clinical and research studies (Ilinca et al. 2019). Malek et al*.* in a large multi-ancestry GWAS of up to 67,162 stroke cases and 454,450 controls discovered 22 new stroke risk loci and validated 10 known stroke loci, bringing the total to 32 loci which are encompassed in the panel (Malik et al. 2018).

### Gene burden testing

The open-source software package Test Rare vAriants with Public Data (TRAPD) was used to perform a gene-based burden testing against public control databases. The software allows for adaptable filtering on various quality and frequency fields to ensure a well-controlled burden test. Gene burden test on our ischemic stroke WES cohort datasets was performed against whole exome or whole genome sequencing data available from approximately 20,230 individuals from the Saudi Human Genome Project (https://shgp.kacst.edu.sa).

### Polygenetic risk score generation

Rare, impactful variants (listed in Supplementary Table S1) identified in stroke genes were included to calculate polygenetic risk scores (PRS) for stroke for each individual study subject to represent the cumulative risk of carrying one or more of these rare variants. (Malik et al. 2018). PRSice-2 software was employed to calculate PRS by setting an equal effect size (beta = 1) for each variant (Choi and O’Reilly 2019). The resulting PRS was standardized for association tests with clinical variables such as age of diagnosis, mRS, and classifications of stroke. It is acknowledged that there may be an under- or over-estimation of the effect size of these rare variants. However, when there is an actual effect, the association, albeit with less accurate estimates, could be detected. The validity of this approach was evident in the initial effort to estimate the combined effect of multiple genetic variants before a more sophisticated statistical approach was developed (Zheng et al. 2008). In addition, as the rare variant PRS was calculated using PRSice-2, it was restricted to the specific set of rare variants that were identified without consideration of other common variants based on LD patterns.

## Results

### Principal component analysis

**Fig. 1:**
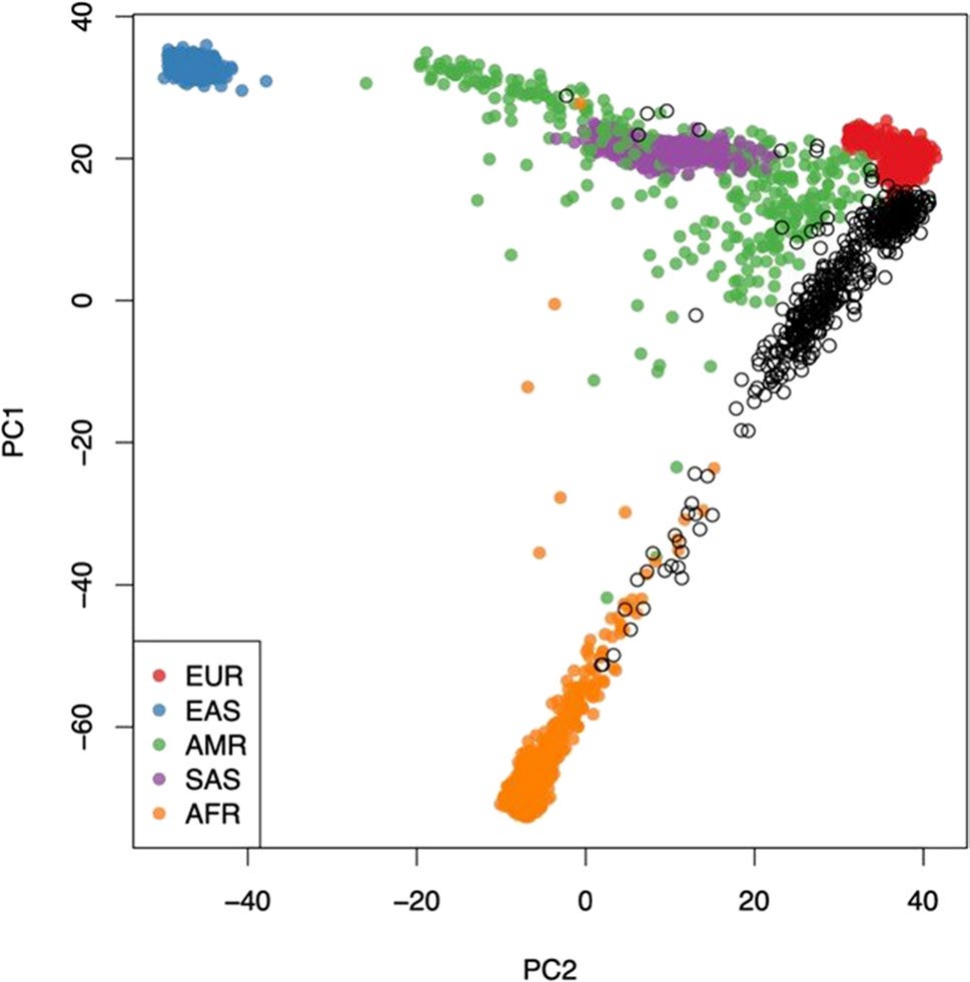
Principal component analysis (PCA) of common SNP genotypes with 1000 genome projects (1KGP) populations reference panel. The x- and y-axes denote the value of two components of PCA (PC1, PC2), with each dot in the figure representing one individual. The color for individuals from 1000 genome projects, Europeans (EUR), East Asians (EAS), Admixed Americans (AMR), South Asians (SAS), and Africans (AFR) are red, blue, green, purple, and orange, respectively. The color for individuals belonging to the Stroke Disease study group is illustrated in black

The common variants of the whole exome sequencing data from the stroke study participants show expected clustering when compared to the world’s major populations using a standard principal component analysis pipeline (Fig. 1). This population stratification data was used to mitigate false attributions in the association analyses components.

### Gene burden testing

We were able to utilize exonic data from 177 genes from the initial set of 214 stroke loci panel (48) after removal of mitochondrial genes; non-exonic regions such as 9p.21 and non-coding RNAs, and genes that had poor quality control and quality assurance filtering data in the Saudi Genome Project control dataset including *ATP7A*, *FLNA*, *GLA*, *GPR143*, *PGAM4,* and *F9*. Focusing on the a priori 177 loci derived from the stroke panel, we identified rare putative impactful variants within the cohort (Supplementary Table S1). The equivalent analysis was performed using the TRAPD burden testing pipeline and we observed that our cohort of 387 ischemic stroke subjects was significantly enriched for impactful alleles in several loci on the a priori stroke panel, compared to the control populations of approximately 20,230 Saudi population-based controls derived from the Saudi Genome Project.

The top 20 most significant signals for gene burden analyses under a dominant model (*p* < 1 × 10^–5^) and 14 signals with *p* < 0.05) under a recessive model are shown on Table 2 with FDR correction and a full list of association signals under both dominant and recessive models are shown for all 177 genes (Supplementary Table S2). Table 2 (top) shows the top 20 genes with *p* < 1 × 10^−5^ under a dominant model (*F13A1*, *NF1*, *ACAD9*, *NOTCH3*, *MYLK*, *SH3PXD2A*, *TSC2*, *ADAMTS13*, *COL4A2*, *APP*, *FOXC1*, *COL1A1*, *TGFBR2*, *PDE4D*, *MYH7*, *DPM1*, *PGM1*, *FCGR2C*, *ZFHX3*, *PDE3A*). Table 2 (top) shows the 14 most significant signals at *p* < 0.05 under a recessive model (*PDE4D*, *KCNQ1*, *TREX1*, *CYP11B1*, *F5*, *HTRA1*, *CACNA1A*, *ZCCHC14*, *NBEAL2*, *FGA*, *PCNT*, *DPM1*, *LOC100505841*, and *SH3PXD2A*). Three genes, *SH3PXD2A*, *PDE4D*, and *DPM1*, were significant under both the dominant and recessive models. Twenty-eight of the genic 32 GWAS loci contained among the 177 genes on the stroke panel were available for analysis and 12 showed gene burden associations at *p* < 0.05 under either dominant or recessive models (*ZCCHC14*, *FGA*, *LOC100505841*, *SH3PXD2A*, *PRPF8*, *PDE3A*, *ZFHX3*, *SH2B3*, *TM4SF4*, *HDAC9–TWIST1*, *SMARCA4*, and *FOXF2).*

**Table 2:** (Top) 20 most significant dominant model signals and (bottom) *p* < 0.05 Recessive model signals from TRAPD gene burden testing in Saudi Ischemic Stroke subjects compared in up to 20,230 individuals from Saudi Human Genome Project

Gene	Heterozygote cases	Homozygote cases	Heterozygote controls	Homozygote controls	P: dominant model	P: recessive model
*F13A1*	10	0	6	0	3.13E − 11	1
*NF1*	11	0	12	2	4.84E − 10	1
*ACAD9*	9	0	11	0	1.52E − 08	1
*NOTCH3*	42	2	411	18	1.77E − 08	0.405288
*MYLK*	31	0	231	13	3.60E − 08	0.170765
*SH3PXD2A*	21	1	126	5	5.16E − 08	0.042198
*TSC2*	48	0	499	15	5.68E − 08	0.791086
*ADAMTS13*	26	0	182	10	1.54E − 07	0.106345
*COL4A2*	27	1	217	9	2.70E − 07	0.435679
*APP*	12	0	44	2	1.19E − 06	1
*FOXC1*	6	1	4	6	1.36E − 06	0.234242
*COL1A1*	21	0	146	3	1.39E − 06	1
*TGFBR2*	5	0	2	0	1.41E − 06	1
*PDE4D*	23	6	253	11	1.58E − 06	2.20E − 06
*MYH7*	7	0	11	0	2.16E − 06	1
*DPM1*	4	1	3	0	3.64E − 06	0.037399
*PGM1*	9	0	25	1	3.88E − 06	0.073402
*FCGR2C*	8	1	26	2	6.40E − 06	0.108062
*ZFHX3* *PDE3A*	105 14	4 0	1791 76	80 3	7.23E − 06 8.31E − 06	0.308726 0.17355
Gene	Heterozygote Cases	Homozygote Cases	Heterozygote Controls	Homozygote Controls	P: Dominant model	P: Recessive model
*PDE4D*	23	6	253	11	1.58E − 06	2.20E − 06
*KCNQ1*	16	1	486	15	0.746853	5.99E − 05
*TREX1*	7	0	126	11	0.293476	0.000408
*CYP11B1*	19	0	209	5	0.001179	0.001053
*F5*	25	1	314	8	0.000606	0.001374
*HTRA1*	2	2	42	0	0.092068	0.001395
*CACNA1A*	20	2	217	14	0.000202	0.009701
*ZCCHC14*	11	2	204	7	0.077487	0.013894
*NBEAL2*	72	2	1840	118	0.623318	0.015113
*FGA*	15	1	253	6	0.053339	0.016894
*PCNT*	79	6	1374	88	0.000112	0.029154
*DPM1*	4	1	3	0	3.64E − 06	0.037399
*LOC100505841*	3	1	39	0	0.075819	0.037399
*SH3PXD2A*	21	1	126	5	5.16E − 08	0.042198

#### Associations of polygenic risk with clinical variables

**Fig. 2:**
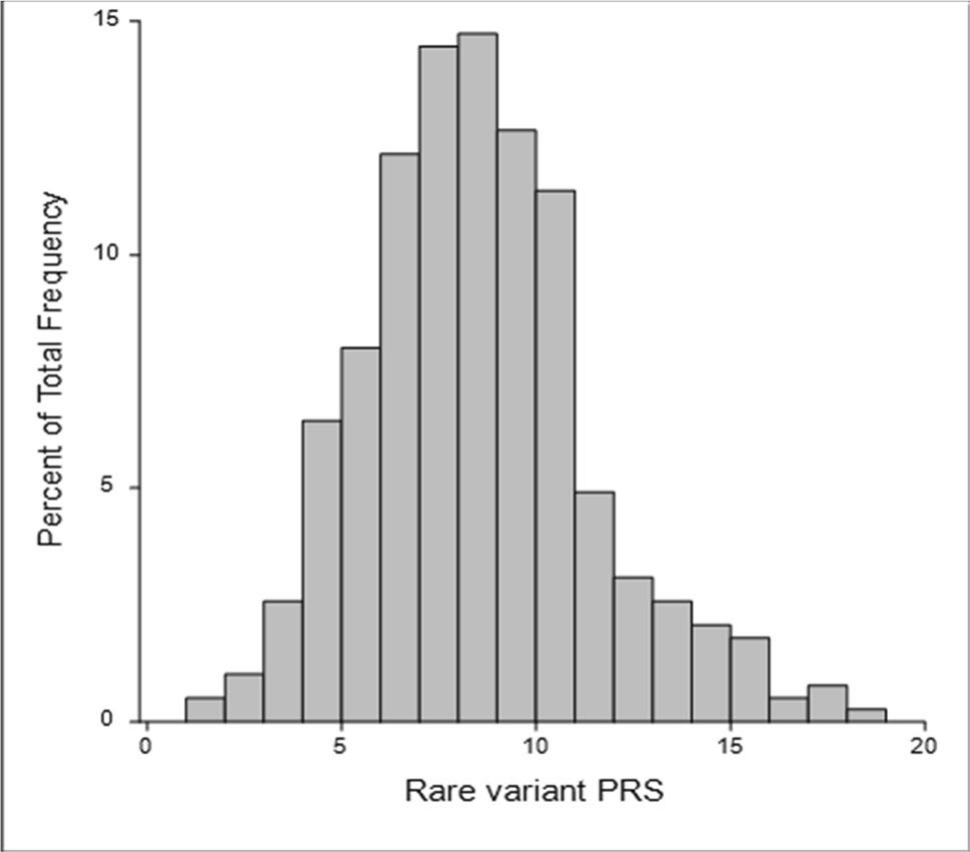
Distribution of rare variant Polygenic Risk Score (PRS) among 387 Saudi Ischemic Stroke subjects. PRS statistics: Mean ± SD = 8.7 ± 3.0; Median[IQR] = 8.6 [6.6,10.5]; Min = 1.53; Max = 18.6

The overall distribution of polygenic risk score (PRS) representing the overall genetic burden from rare, impactful variants in stroke genes among all 387 ischemic stroke study participants subjected to whole exome sequencing deviated from normality and was right-skewed (Fig. 2) with median [IQR] of 8.61 [6.59,10.53], minimum of 1.53, and maximum of 18.75. PRS was not associated with age of diagnosis, ischemia diagnosis, and classification of stroke such as large artery atherosclerosis, cardio aortic embolism, and small artery occlusion (Table 3). However, a significant association of PRS and mRS, which measures the degree of disability post-stroke, was identified (regression coefficient (95%CI) = 0.15 (0.03–0.27), *p* = 0.02). PRS was found to increase the risk of developing greater disability post-stroke event (mRS > 3), when compared to patients with lower mRS (mRS ≤ 3) (OR (95%CI) = 1.79 (1.29–2.49), *p* = 0.0005) (Table 3).

**Table 3:** Association of clinical phenotypes with polygenic risk score (PRS) constructed with rare impactful variants in stroke genes. The PRS was standardized to mean at 0 and SD of 1

Continuous phenotype	Regression coefficient (95%CI)	*p*-value
Age of Diagnosis	1.15 (− 0.43–2.72)	0.15
Modified Rankin Scale (mRS)	0.15 (0.03–0.27)	0.02
Dichotomized phenotype	OR (95%CI)	*p*-value
High mRS (4/5/6, *N* = 35) vs low mRS (0/1, N = 95)	1.56 (1.09–2.23)	0.015
High mRS (4/5/6, *N* = 35) vs lower mRS (0/1/2/3, *N* = 351)	1.79 (1.29–2.49)	0.0005
Diagnosis (= ischemia)	1.14 (0.89–1.47)	0.31
Large artery atherosclerosis	1.00 (0.75–1.34)	0.98
Cardio aortic embolism	0.87 (0.71–1.07)	0.2
Small artery occlusion	1.15 (–0.43–2.72)	0.15

**Fig. 3:**
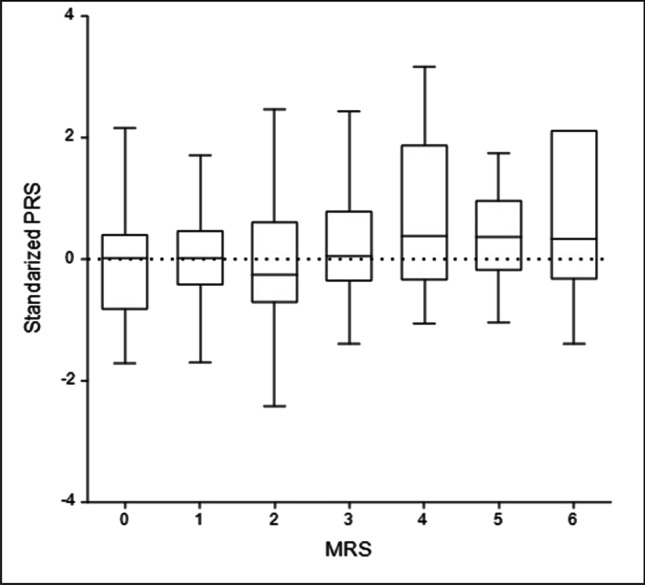
Box-whisker plot of standardized Stroke Polygenic risk score in a Saudi Stroke Cohort by Modified Rankin Scale (mRS) groups. The standardized Polygenic Risk Score (PRS) is illustrated on the y-axis with the Modified Rankin Scale (mRS) groups shown on the x-axis

Stroke subjects with mRSs above 3 were found to carry greater cumulative genetic risk from rare variants in stroke genes (standardized PRS mean > 0) compared to the population average (standardized PRS mean = 0). However, patients with mRS of 3 or lower had lower cumulative genetic risk from rare variants in stroke genes, with the means of standardized PRS at or lower than 0 (Fig. 3).

## Discussion

In this study, WES was performed on 387 individuals diagnosed with ischemic stroke. We initially focused on known, or putative, pathogenic exonic variants evident in 177 genes prioritized from a comprehensive panel of loci associated with monogenic causes of stroke as well as recent GWAS statistically significant signals (Ilinca et al. 2019). This panel has been used in exome sequencing interpretation of individuals from multi-incident stroke families to generate and assess putative pathogenic variants amongst the probands and wider family members (Ilinca et al. 2020). It has also been utilized in cerebral small vessel disease (CSVD) which revealed putative pathogenic variants in multiple loci (Monkare et al. 2022). Using the TRAPD gene burden testing pipeline, filtered on our list of 177 genes, we found significant enrichment for pathogenic or likely pathogenic stroke variants in numerous genes in our cohort using datasets from the Saudi Genome Project as controls.

A number of the top signals in our study are outlined below. The coagulation factor XIII A chain (*F13A1*) gene encodes the coagulation factor that is the last component activated in the blood coagulation cascade. Factor XIII deficiency is typically classified into two categories: type I deficiency, characterized by the lack of both the A and B subunits; and type II deficiency, characterized by the lack of the A subunit alone. These defects can result in defective wound healing and a tendency for lifelong bleeding. A review of common variants association analyses of *F13A1* with stroke phenotypes has shown mixed findings. However, a large Women’s Health Initiative (WHI) study of 2,045 of post-menopausal women, significant risk was observed for both ischemic stroke and for combined ischemic and hemorrhagic between two common *F13A1* variants and with hormone replacement therapy (Huang et al. 2012). A Dutch study also showed significant associations between common variants in *F13A1* with ischemic stroke in young women with the effect more pronounced with oral contraception use (Pruissen et al. 2008).

Neurofibromin 1 (*NF1*) gene encodes a negative regulator of the Ras signal transduction pathway and a number of *NF1* mutations have been linked to neurofibromatosis type 1 which is commonly associated with malignant tumors and cardiovascular or cerebrovascular complications (Napolitano et al. 2022). Neurofibromatosis type 1 also increases the risk of vasculopathies and arterial wall weakness and can lead to complications such as hemorrhagic stroke, ischemic stroke, and multi-domain cognitive impairment (Napolitano et al. 2022).

Acyl-CoA dehydrogenase family member 9 (ACAD9) encodes a member of the acyl-CoA dehydrogenase family, a family of proteins that are localized in the mitochondria and are involved in beta-oxidation of fatty acyl-CoA. Mutations in this gene cause acyl-CoA dehydrogenase family member type 9 deficiency which often results in intellectual disability and neurologic dysfunction. A homozygous variant in ACAD9 was identified in the proband of a Swedish family where family members reported stroke with intracerebral bleeding and progressive muscle and heart failure (Ilinca et al. 2020). A case report of a death of a teenager with ACAD9 deficiency with Reye-like episode and cerebellar stroke has also been reported (Huang et al. 2012).

Phosphodiesterase 4D (*PDE4D*) encodes up to 9 different isoforms whose functional proteins degrade the second messenger Cyclic adenosine monophosphate (cAMP), a key signal transduction molecule in multiple cell types, including vascular cells (Munshi & Kaul 2008). Numerous case–control studies have been performed to assess the association between *PDE4D* variants and ischemic stroke risk among different ancestral populations. In particular, the so-called “SNP83” (rs966221) association with has been robust in many of these studies (Munshi & Kaul 2008; Xu et al. 2010). *PDE4D* is associated with inflammation and reduced PDE4D is thought to increase the risk of atrial fibrillation, which in turn increases stroke risk (Jørgensen et al. 2015). Several studies have shown a robust association of *PDE4D* variants with ischemic stroke in young individuals (Yue et al. 2019).

Potassium voltage-gated channel subfamily Q member 1 (*KCNQ1*) encodes a voltage-gated potassium channel protein which is required for the repolarization phase of the cardiac action potential and forms multimers with KCNE1, KCNE3 and potassium channel proteins. Mutations in *KCNQ1* are associated with familial atrial fibrillation and hereditary long QT syndrome 1 which can impact stroke risk (Jørgensen et al. 2015; Lavy et al. 1974). An increased genetic burden of rare deleterious *KCNQ1* variants in Polish subjects with large‑vessel ischemic stroke were identified using second-generation sequencing (Janicki et al. 2019).

Three prime repair exonuclease 1 (*TREX1*) encodes a nuclear protein with 3′ exonuclease activity and play a role in DNA repair and serve as a proofreading function for DNA polymerase. Mutations in this gene result in diseases of the immune system including Aicardi-Goutieres syndrome and other autoimmune-type diseases which can put subjects at a higher risk of ischemic stroke. Uemura 2023 investigating the prevalence of Mendelian stroke genes mutations in monogenic cerebral small vessel stroke patients aged 55 years or younger from a Japanese stroke registry identified a *TREX1* pathogenic genetic variants in one stroke subject (Uemura et al. 2023). A large Mendelian Stroke Consortium also identified pathogenic clinical variants in *TREX1* (Grami et al. 2020).

The distribution of the polygenic risk score analyses showed that the stroke subjects in this Saudi cohort each, on average, carry over 8 rare, impactful variants. PRS analyses performed on a weighted cumulative risk from rare, impactful variants among ischemic stroke participants with high Modified Rankin Scale (mRS), i.e., 4, 5, 6 versus mRS 0, 1, 2, 3 showed a significant association (OR (95%CI) = 1.79 (1.29–2.49), *p* = 0.0005). Determining the potential mRS cutoffs to use for clinical significance within a highly consanguineous population like that in Saudi Arabia may yield translational value, such as risk stratification, especially with the additional of common and rare variants to the PRS from ongoing stroke genome-wide studies.

In Saudi Arabia, undetected or untreated vascular disease is a significant health and financial burden^,^(Walli-Attaei et al. 2020a, b; Bindawas & Vennu 2016). There is a compelling need for implementation of primary and secondary stroke prevention strategies in Saudi Arabia due to an increasing incidence rate with the mortality rate projected to almost double by 2030 (Robert et al. 2018; Bindawas & Vennu 2016). In this study using gene-burden analyses in 387 Saudi Arabian ischemic stroke subjects and 20,230 controls from the Saudi Human Genome Project, we observed significant associations in dozens of loci under autosomal dominant and/or recessive modelling. Stroke subjects with Modified Rankin Scale (mRSs) above 3 were observed to have a greater cumulative PRS from rare variants in stroke genes when compared to the population average. Interestingly, stroke subjects with mRS of 3 or lower had lower cumulative genetic risk from rare variants in stroke genes (Alokley and Albakr 2022). Determining the potential mRS cutoffs to use for clinical significance may allow risk stratification of this population.

## Supplementary Information

Below is the link to the electronic supplementary material.

Supplementary file1 (XLSX 874 KB)

Supplementary file2 (DOCX 43 KB)

## Data Availability

The datasets generated during the current study are available in the European Variation Archive (EVA) repository (https://www.ebi.ac.uk/ebisearch/search?query=PRJEB59227&submit=Search&db=allebi&requestFrom=global-masthead), under the title *Whole‐Exome Sequencing Analyses in a Saudi Ischemic Stroke Cohort Reveal Association* Signals and shows Polygenic Risk Scores are related to Modified Rankin Scale Risk with accession number PRJEB59227. All requests for data can be sent to the corresponding author (AKA) and verified academic investigators will be granted full access.
