# Supplementary material for: Whole‐exome sequencing analyses in a Saudi Ischemic Stroke Cohort reveal association signals, and shows polygenic risk scores are related to Modified Rankin Scale Risk
Source: Funct Integr Genomics. 2023 Mar 27;23(2):102. doi: 10.1007/s10142-023-01039-7 (PMC10042957; doi:10.1007/s10142-023-01039-7)
Supplement: Supplementary file 2 — Supplementary file2 (DOCX 43 KB) [file 10142_2023_1039_MOESM2_ESM.docx]

**Article title**: Whole‐Exome Sequencing Analyses in a Saudi Ischemic Stroke Cohort Reveal Association Signals, and shows Polygenic Risk Scores are related to modified Rankin Scale Risk

**Journal Name:** Neuromolecular Medicine

**Author Names**^: 1^Fahad A. Alkhamis MD, ^1^Majed M. Alabdali MD, ^1^Abdulla A Alsulaiman MD, ^1^Abdullah S. Alamri MD, ^2^Rudaynah Alali MD, ^3^Mohammed S. Akhtar PhD, ^4^Sadiq A. Alsalman MD,^3^Cyril Cyrus PhD, ^1^Aishah I Albalr MD, ^1^Anas S. Alduhalan MD, ^5^Mohamed Abouelhoda PhD, ^5^Khaldoun Al- Romaih PhD, ^6^Divya Gandla PhD , ^6^Bao-Li Loza PhD, ^6^Brendan Keating PhD, ^3^Amein A. Al-Ali PhD.

**Affiliation:** ^1^Department of Neurology, King Fahd Hospital of The University, Imam Abdulrahman Bin Faisal University, Dammam 31441, Saudi Arabia;^3^Department of Internal Medicine, King Fahd Hospital of The University, Imam Abdulrahman Bin Faisal University, Dammam 31441, Saudi Arabia;^3^Department of clinical Biochemistry, College of Medicine, Imam Abdulrahman Bin Faisal University, Dammam 31441, Saudi Arabia;^4^Department of Neurology, King Fahd Hospital, Alhafof , Saudi Arabia ;^5^Centre for Genomic Medicine, KFSH&RC, Riyadh, 11211,  Saudi Arabia; ^6^Department of Surgery, Perelman School of Medicine, University of Pennsylvania, Pennsylvania, PA 19104, USA.

**Corresponding author email:** aalali@iau.edu.sa

**Supplementary Table 2:** Association Significances under Dominant and Recessive Modeling after TRAPD Gene Burden Testing) in 387 Saudi stroke subjects using 177 Stroke related genes versus up to 20,230 individuals from Saudi Human Genome Project

| **Gene** | Heterozygote Cases | Homozygote Cases | Heterozygote Controls | Homozygote Controls | P: Dominant model | P: Recessive model |
| --- | --- | --- | --- | --- | --- | --- |
| *ABCA1* | 50 | 1 | 822 | 38 | 0.00218144 | 0.77997586 |
| *ABCC6* | 44 | 1 | 584 | 16 | 3.46E-05 | 0.05391427 |
| *ABCG5* | 14 | 0 | 401 | 9 | 0.72325561 | 0.08537 |
| *ABCG8* | 20 | 1 | 389 | 16 | 0.11905697 | 0.70523344 |
| *ABO* | 15 | 0 | 318 | 13 | 0.31471078 | 0.59981044 |
| *ACAD9* | 9 | 0 | 11 | 0 | 1.52E-08 | 1 |
| *ACP5* | 15 | 0 | 296 | 14 | 0.23599435 | 1 |
| *ACVRL1* | 7 | 0 | 89 | 4 | 0.08047663 | 1 |
| *ADAMTS13* | 26 | 0 | 182 | 10 | 1.54E-07 | 0.10634474 |
| *ADIPOQ* | 16 | 0 | 378 | 20 | 0.48313215 | 1 |
| *ANK2* | 51 | 0 | 1548 | 68 | 0.95530578 | 0.9798969 |
| *APOB* | 85 | 0 | 1593 | 56 | 0.00406891 | 0.43781125 |
| *APP* | 12 | 0 | 44 | 2 | 1.19E-06 | 1 |
| *ASS1* | 4 | 0 | 2 | 1 | 6.16E-05 | 1 |
| *ATP7A* | 0 | 0 | 217 | 120 | 1 | 1 |
| *B4GALT1* | 5 | 0 | 28 | 1 | 0.00811122 | 1 |
| *C1orf167* | 11 | 0 | 114 | 2 | 0.00795721 | 0.14142884 |
| *C1R* | 6 | 0 | 24 | 0 | 0.00072859 | 1 |
| *CACNA1A* | 20 | 2 | 217 | 14 | 0.00020164 | 0.00970078 |
| *CASZ1* | 67 | 2 | 1631 | 72 | 0.37477837 | 0.99673954 |
| *CBS* | 9 | 0 | 66 | 5 | 0.00289265 | 1 |
| *CD59* | 2 | 0 | 24 | 0 | 0.25380268 | 1 |
| *CDK6* | 1 | 0 | 53 | 8 | 0.90654805 | 1 |
| *COG6* | 9 | 0 | 257 | 11 | 0.71418973 | 0.51559935 |
| *COL1A1* | 21 | 0 | 146 | 3 | 1.39E-06 | 1 |
| *COL3A1* | 18 | 0 | 433 | 10 | 0.46001932 | 0.68181117 |
| *COL4A1* | 13 | 0 | 201 | 8 | 0.07336953 | 1 |
| *COL4A2* | 27 | 1 | 217 | 9 | 2.70E-07 | 0.43567872 |
| *COL5A1* | 31 | 1 | 447 | 13 | 0.00158743 | 0.7269357 |
| *COL5A2* | 28 | 0 | 586 | 40 | 0.2532534 | 0.78292235 |
| *CPS1* | 30 | 0 | 640 | 27 | 0.2346771 | 1 |
| *CST3* | 1 | 0 | 0 | 1 | 0.07340189 | 1 |
| *CSTF2T* | 5 | 0 | 16 | 3 | 0.0016824 | 1 |
| *CTSA* | 3 | 0 | 17 | 0 | 0.03689535 | 1 |
| *CUL3* | 0 | 0 | 0 | 0 | 1 | 1 |
| *CYP11B1* | 19 | 0 | 209 | 5 | 0.00117868 | 0.00105262 |
| *DLD* | 4 | 0 | 10 | 0 | 0.0014305 | 1 |
| *DPM1* | 4 | 1 | 3 | 0 | 3.64E-06 | 0.03739853 |
| *DYRK1B* | 9 | 1 | 71 | 4 | 0.00122073 | 0.2044735 |
| *EFEMP2* | 2 | 0 | 79 | 8 | 0.85125955 | 1 |
| *ELN* | 41 | 1 | 920 | 56 | 0.27085611 | 0.9100415 |
| *ENG* | 18 | 1 | 452 | 19 | 0.46939918 | 0.46947663 |
| *ENPP1* | 9 | 0 | 88 | 2 | 0.01163969 | 1 |
| *EPAS1* | 16 | 0 | 169 | 15 | 0.00340549 | 1 |
| *EPOR* | 17 | 0 | 340 | 13 | 0.22321607 | 1 |
| *ESCO2* | 12 | 1 | 156 | 4 | 0.01298459 | 0.23424228 |
| *EVI2A* | 15 | 0 | 391 | 27 | 0.65832645 | 1 |
| *EVI2B* | 1 | 0 | 4 | 0 | 0.17355056 | 1 |
| *F10* | 4 | 0 | 71 | 3 | 0.33370909 | 0.17355056 |
| *F13A1* | 10 | 0 | 6 | 0 | 3.13E-11 | 1 |
| *F13B* | 5 | 0 | 11 | 0 | 0.00022131 | 1 |
| *F2* | 8 | 0 | 61 | 1 | 0.00442789 | 1 |
| *F5* | 25 | 1 | 314 | 8 | 0.00060593 | 0.00137351 |
| *F7* | 5 | 0 | 74 | 1 | 0.17941837 | 1 |
| *F9* | 0 | 0 | 0 | 0 | 1 | 1 |
| *FBLN5* | 11 | 0 | 151 | 8 | 0.05413648 | 1 |
| *FBN1* | 13 | 0 | 75 | 3 | 3.10E-05 | 0.17355056 |
| *FCGR2C* | 8 | 1 | 26 | 2 | 6.40E-06 | 0.108062 |
| *FGA* | 15 | 1 | 253 | 6 | 0.05333943 | 0.01689356 |
| *FGB* | 13 | 0 | 172 | 10 | 0.03134434 | 1 |
| *FGG* | 19 | 1 | 440 | 29 | 0.37185563 | 0.84620018 |
| *FLNA* | 0 | 0 | 201 | 136 | 1 | 1 |
| *FOXC1* | 6 | 1 | 4 | 6 | 1.36E-06 | 0.23424228 |
| *FOXF2* | 5 | 0 | 15 | 5 | 0.00203936 | 1 |
| *FURIN* | 36 | 2 | 809 | 51 | 0.23205199 | 0.83200153 |
| *GAA* | 32 | 1 | 393 | 15 | 0.00011034 | 0.13288729 |
| *GCDH* | 1 | 0 | 2 | 0 | 0.108062 | 1 |
| *GFI1B* | 8 | 0 | 36 | 0 | 0.00019337 | 1 |
| *GGCX* | 24 | 2 | 398 | 19 | 0.01522147 | 0.40528836 |
| *GLA* | 0 | 0 | 0 | 0 | 1 | 1 |
| *GP1BA* | 18 | 3 | 227 | 21 | 0.00115552 | 0.09270102 |
| *GPR143* | 0 | 0 | 37 | 27 | 1 | 1 |
| *GYS1* | 9 | 0 | 43 | 1 | 0.00013418 | 1 |
| *HBB* | 6 | 0 | 58 | 2 | 0.03633505 | 1 |
| *HCFC2* | 1 | 0 | 0 | 0 | 0.03739853 | 1 |
| *HDAC9* | 18 | 0 | 174 | 6 | 0.00044409 | 1 |
| *HSD11B2* | 2 | 0 | 16 | 2 | 0.17076462 | 1 |
| *HTRA1* | 2 | 2 | 42 | 0 | 0.09206812 | 0.00139517 |
| *IL1RN* | 7 | 1 | 135 | 6 | 0.19471886 | 0.29048767 |
| *ISCU* | 8 | 0 | 96 | 3 | 0.04694414 | 0.17355056 |
| *ITM2B* | 2 | 0 | 17 | 1 | 0.17076462 | 1 |
| *IVD* | 16 | 0 | 193 | 12 | 0.00879678 | 1 |
| *JAG1* | 22 | 0 | 272 | 11 | 0.00244044 | 0.17076462 |
| *JAK2* | 15 | 0 | 148 | 4 | 0.00147322 | 1 |
| *JAM3* | 3 | 0 | 13 | 0 | 0.02022644 | 1 |
| *KCNA5* | 9 | 0 | 60 | 3 | 0.00137933 | 0.14142884 |
| *KCNE2* | 12 | 1 | 521 | 27 | 0.97984743 | 0.87778387 |
| *KCNJ2* | 2 | 0 | 0 | 0 | 0.00139517 | 1 |
| *KCNK3* | 15 | 0 | 247 | 16 | 0.09866139 | 1 |
| *KCNQ1* | 16 | 1 | 486 | 15 | 0.74685263 | 5.99E-05 |
| *KIAA1257* | 1 | 0 | 15 | 0 | 0.45681414 | 1 |
| *KLHL3* | 5 | 0 | 24 | 1 | 0.00469307 | 1 |
| *KNG1* | 24 | 0 | 800 | 34 | 0.95146337 | 0.89312917 |
| *LDLR* | 9 | 0 | 163 | 4 | 0.21343393 | 1 |
| *LMNA* | 4 | 0 | 14 | 1 | 0.00477675 | 1 |
| *LOC100505841* | 3 | 1 | 39 | 0 | 0.07581888 | 0.03739853 |
| *LRCH1* | 7 | 1 | 121 | 4 | 0.12527586 | 0.2044735 |
| *MFAP5* | 19 | 1 | 439 | 26 | 0.35789856 | 0.82747691 |
| *MFN2* | 41 | 3 | 992 | 66 | 0.34407923 | 0.95371426 |
| *MGAT2* | 17 | 1 | 485 | 22 | 0.68398974 | 0.83395783 |
| *MMACHC* | 9 | 0 | 112 | 1 | 0.03912463 | 1 |
| *MMP12* | 44 | 2 | 943 | 63 | 0.14594747 | 0.93336966 |
| *MPI* | 36 | 3 | 955 | 100 | 0.65151008 | 0.93910803 |
| *MTHFR* | 14 | 0 | 203 | 10 | 0.04575747 | 0.43567872 |
| *MYH11* | 46 | 0 | 710 | 34 | 0.00167444 | 0.64483553 |
| *MYH7* | 7 | 0 | 11 | 0 | 2.16E-06 | 1 |
| *MYLK* | 31 | 0 | 231 | 13 | 3.60E-08 | 0.17076462 |
| *NBEAL2* | 72 | 2 | 1840 | 118 | 0.62331849 | 0.0151133 |
| *NEURL2* | 4 | 1 | 116 | 4 | 0.50384634 | 0.2044735 |
| *NF1* | 11 | 0 | 12 | 2 | 4.84E-10 | 1 |
| *NOTCH1* | 41 | 0 | 613 | 17 | 0.00122694 | 0.17842054 |
| *NOTCH3* | 42 | 2 | 411 | 18 | 1.77E-08 | 0.40528836 |
| *NPPA* | 3 | 0 | 76 | 3 | 0.5969522 | 1 |
| *NR3C2* | 8 | 0 | 22 | 1 | 1.32E-05 | 1 |
| *NUP155* | 14 | 0 | 243 | 14 | 0.13849015 | 0.55121943 |
| *OMG* | 5 | 0 | 26 | 2 | 0.00713231 | 1 |
| *PCCA* | 1 | 0 | 6 | 0 | 0.23424228 | 1 |
| *PCCB* | 17 | 3 | 385 | 17 | 0.16413096 | 0.14155277 |
| *PCNT* | 79 | 6 | 1374 | 88 | 0.00011227 | 0.02915356 |
| *PDE3A* | 14 | 0 | 76 | 3 | 8.31E-06 | 0.17355056 |
| *PDE4D* | 23 | 6 | 253 | 11 | 1.58E-06 | 2.20E-06 |
| *PGAM4* | 0 | 0 | 10 | 10 | 1 | 1 |
| *PGM1* | 9 | 0 | 25 | 1 | 3.88E-06 | 0.07340189 |
| *PITX2* | 2 | 0 | 17 | 0 | 0.15737769 | 1 |
| *PKD1* | 58 | 4 | 856 | 45 | 1.30E-05 | 0.05049291 |
| *PLG* | 12 | 0 | 108 | 6 | 0.00263335 | 1 |
| *PLOD1* | 29 | 0 | 437 | 13 | 0.00711006 | 0.14155277 |
| *PLOD3* | 29 | 0 | 626 | 32 | 0.2734288 | 1 |
| *PMF1* | 1 | 0 | 6 | 0 | 0.23424228 | 1 |
| *PMM2* | 4 | 0 | 84 | 4 | 0.45273019 | 1 |
| *PRKG1* | 5 | 0 | 17 | 0 | 0.00110853 | 1 |
| *PROS1* | 16 | 0 | 144 | 28 | 0.00182392 | 1 |
| *PRPF8* | 5 | 1 | 10 | 2 | 3.33E-05 | 0.108062 |
| *PTPN11* | 2 | 0 | 9 | 0 | 0.06138347 | 1 |
| *RASA1* | 14 | 0 | 156 | 9 | 0.00725018 | 1 |
| *RASGRP2* | 6 | 0 | 125 | 2 | 0.37929312 | 1 |
| *REXO4* | 4 | 0 | 38 | 0 | 0.07075339 | 1 |
| *RGS7* | 2 | 0 | 67 | 4 | 0.76367011 | 1 |
| *SAG* | 16 | 0 | 339 | 16 | 0.31404398 | 1 |
| *SAMHD1* | 7 | 0 | 67 | 1 | 0.02171787 | 1 |
| *SCN1B* | 7 | 2 | 265 | 11 | 0.7448008 | 0.17076462 |
| *SCN2B* | 3 | 0 | 0 | 0 | 5.19E-05 | 1 |
| *SCN5A* | 37 | 0 | 480 | 17 | 0.00020504 | 0.79108641 |
| *SCNN1B* | 33 | 0 | 911 | 44 | 0.78115541 | 1 |
| *SCNN1G* | 3 | 0 | 4 | 0 | 0.00162358 | 1 |
| *SERPINC1* | 5 | 0 | 115 | 4 | 0.49671996 | 1 |
| *SERPINE1* | 17 | 1 | 459 | 21 | 0.59497926 | 0.49397712 |
| *SH2B3* | 38 | 5 | 658 | 93 | 0.00860568 | 0.43972058 |
| *SH3PXD2A* | 21 | 1 | 126 | 5 | 5.16E-08 | 0.04219756 |
| *SLC19A2* | 9 | 0 | 81 | 5 | 0.0089943 | 1 |
| *SLC22A7* | 10 | 0 | 146 | 6 | 0.08240642 | 1 |
| *SLC2A10* | 8 | 0 | 202 | 7 | 0.56757302 | 1 |
| *SLC44A2* | 16 | 0 | 398 | 9 | 0.51849854 | 1 |
| *SMAD3* | 2 | 0 | 14 | 0 | 0.11864736 | 1 |
| *SMAD4* | 3 | 0 | 22 | 0 | 0.06498065 | 1 |
| *SMARCA4* | 9 | 0 | 58 | 2 | 0.00101337 | 1 |
| *SMARCAL1* | 7 | 0 | 107 | 2 | 0.14377124 | 1 |
| *SPARC* | 7 | 0 | 108 | 3 | 0.15300834 | 1 |
| *STAT1* | 1 | 0 | 2 | 0 | 0.108062 | 1 |
| *STIM1* | 8 | 0 | 70 | 4 | 0.01136584 | 0.17355056 |
| *TBX20* | 4 | 0 | 55 | 2 | 0.1935831 | 1 |
| *TBX3* | 7 | 0 | 77 | 6 | 0.05116974 | 1 |
| *TGFB2* | 3 | 0 | 9 | 0 | 0.00887592 | 1 |
| *TGFB3* | 2 | 0 | 24 | 0 | 0.25380268 | 1 |
| *TGFBR1* | 0 | 0 | 6 | 0 | 1 | 1 |
| *TGFBR2* | 5 | 0 | 2 | 0 | 1.41E-06 | 1 |
| *THBD* | 10 | 0 | 117 | 5 | 0.02611948 | 1 |
| *TM4SF4* | 3 | 0 | 0 | 0 | 5.19E-05 | 1 |
| *TREX1* | 7 | 0 | 126 | 11 | 0.29347568 | 0.00040806 |
| *TSC1* | 5 | 1 | 51 | 5 | 0.02779769 | 0.2044735 |
| *TSC2* | 48 | 0 | 499 | 15 | 5.68E-08 | 0.79108641 |
| *TSPAN2* | 1 | 0 | 1 | 0 | 0.07340189 | 1 |
| *TTR* | 5 | 0 | 191 | 4 | 0.87374538 | 1 |
| *VHL* | 3 | 0 | 80 | 3 | 0.62933218 | 1 |
| *VWF* | 41 | 0 | 532 | 14 | 7.70E-05 | 0.23642839 |
| *WFS1* | 62 | 1 | 929 | 45 | 6.49E-05 | 0.41293285 |
| *WNT2B* | 8 | 4 | 123 | 91 | 0.14076567 | 0.49377505 |
| *YY1AP1* | 13 | 0 | 242 | 5 | 0.17643936 | 0.36722321 |
| *ZCCHC14* | 11 | 2 | 204 | 7 | 0.07748654 | 0.01389357 |
| *ZFHX3* | 105 | 4 | 1791 | 80 | 7.23E-06 | 0.30872587 |
